# Does Schizophrenia Itself Cause Obesity?

**DOI:** 10.3389/fpsyt.2022.934384

**Published:** 2022-06-23

**Authors:** Jiaquan Liang, Yanshan Cai, Xiongyan Xue, Xiaoling Li, Zaifang Li, Caixia Xu, Guojun Xie, Yang Yu

**Affiliations:** Department of Psychiatry, The Third People’s Hospital of Foshan, Foshan, China

**Keywords:** metabolic indexes, obesity, overweight, body mass index, schizophrenia

## Abstract

**Background:**

Schizophrenia (SC) is considered the most serious of all mental disorders. Some antipsychotics are associated with weight gain and metabolic abnormalities. Whether SC itself causes obesity remains uncertain.

**Methods:**

We collected 185 first-episode drug-naive SC and 59 healthy controls (HCs) from the Third People’s Hospital of Foshan, Guangdong, China, and distinguished their course of disease in order to understand the body mass index (BMI) and body fat metabolism of SC.

**Results:**

We found that excluding the drug factors, the longer the course of SC, the more obvious the increase of BMI and the higher the proportion of obesity. BMI was positively correlated with age, course of disease, fasting blood glucose (FBG), low-density lipoprotein (LDL), triglyceride (TG), and total cholesterol (TC), and negatively correlated with high-density lipoprotein (HDL). The results of regression analysis were further proof that age (*B* = 0.094, *p* < 0.001), duration (B = 0.081, *p* = 0.002), FBG (*B* = 0.987, *p* = 0.004), and TG (*B* = 0.918, *p* = 0.002) were the risk factors for the increase of BMI. HDL (B = –2.875, *p* < 0.001) was the protective factor.

**Conclusion:**

SC itself can increase BMI and easily lead to obesity. We should pay more attention to the monitoring of blood metabolism indicators, so as to reduce the risk of obesity and improve the quality of life of patients.

## Introduction

Schizophrenia (SC) is considered the most serious of all mental disorders (1, 2). Many patients with SC do not fully recover, and even among those with a good prognosis, the disease changes their lives, including social isolation, stigma, and reduced likelihood of finding a companion (3, 4). Poor eating habits, weight gain, smoking, and substance abuse can reduce life expectancy by 13–15 years (5, 6). Antipsychotics are commonly used to treat many different mental disorders (7). Some drugs are related to weight gain and metabolic abnormalities (8–10). Therefore, some people believe that obesity in SC is mainly caused by antipsychotics (11). However, whether SC itself causes obesity remains uncertain. Hence, we collected the first-episode drug-naive SC from Foshan, Guangdong, China, and distinguished their course of disease, in order to understand the body mass index (BMI) and body fat metabolism of the SC.

## Materials and Methods

### Participants

The outpatients and inpatients with SC, who met the diagnostic criteria of the *Diagnostic and Statistical Manual of Mental Disorders*, Fourth Edition (DSM-IV) from August 2016 to September 2021 in the Department of Psychiatry, The Third People’s Hospital of Foshan, Guangdong, People’s Republic of China, were included. According to the course of disease, they were divided into groups A (duration < 6 months), B (6 months ≤ duration<24 months), and C (duration ≥ 24 months).

Inclusion criteria were as follows: (1) SC: ➀ 18–45 years old (in order to avoid physical diseases caused by aging); ➁ years of education ≥ 6; ➂ Han nationality; ➃ before test, did not take any antipsychotics, antidepressants, mood stabilizers, sedatives, etc.; (2) healthy control (HC): volunteers recruited through advertising in Foshan from March 2020 to December 2021; ➀ 18–45 years old; ➁ no history or family history of psychosis; ➂ Han nationality; ➃ years of education ≥ 6; ➄ gender, age, and education were matched with the patient group.

Exclusion criteria were as follows: ① comorbidity other mental disorders, including intellectual disability or other cognitive impairment; ② patients with diabetes, hypertension, severe kidney, liver function damage, cardiac insufficiency, etc.; ③ those who did not cooperate with venous blood drawing due to phobia, etc.; ④ smoking habits (≥ 1 cigarette per day) or drinking habits (≥ 1 unit alcohol per week); 1 unit alcohol = 480–600 ml of beer = 350 ml of low alcohol liquor or red wine, yellow wine = 50 ml of high spirits (40° or more).

### Assessment and Blood Sampling

Details such as names, gender, and age were collected through the interviews from subjects who were willing to participate, after receiving the signed informed consent form. Then, the weight, height, and BMI of these subjects were measured with Automatic Measuring Stadiometer BSM370 (https://smitechasia.com). The positive and negative syndrome scale (PANSS) was used to assess the extent of the patient’s mental symptoms.

According to the diagnostic criteria of overweight and obesity proposed by the China Obesity working group, “BMI<24 kg/m^2^” was defined as non-obesity, “24 kg/m^2^ ≤ BMI<28 kg/m^2^” was defined as overweight, and “BMI ≥ 28 kg/m^2^ as obesity” was defined as obesity (12).

Subjects were instructed to be on fasting for more than 8 h before drawing the venous blood and the night before blood drawing, to maintain a normal diet, not to drink any alcohol or coffee after dinner, and to avoid strenuous exercise. FBG, HDL, LDL, TG, and TC would be recorded in the subject’s clinical data sheet for subsequent data analysis.

### Statistical Methods

We used the Statistical Product and Service Solutions 19 software^1^ to analyze the data. Chi-square test was used to compare the differences of general demographic parameters. The blood metabolic indexes, which correspond to normal distribution, were compared by one-way ANOVA and non-parametric data were compared by Kruskal-Wallis test. The relationships of BMI and variable indexes were analyzed by Pearson correlation. Multiple linear regression was used to analyze the influencing factors of BMI.

## Results

### Comparison of Demographic Characteristics and Metabolic Indexes

There were 244 participants in this study, including HC (*n* = 59), A (*n* = 92), B (*n* = 45), and C (*n* = 48), while 10 subjects were excluded due to consumption of breakfast before drawing blood or due to not showing interest in performing blood test.

**FIGURE 1:**
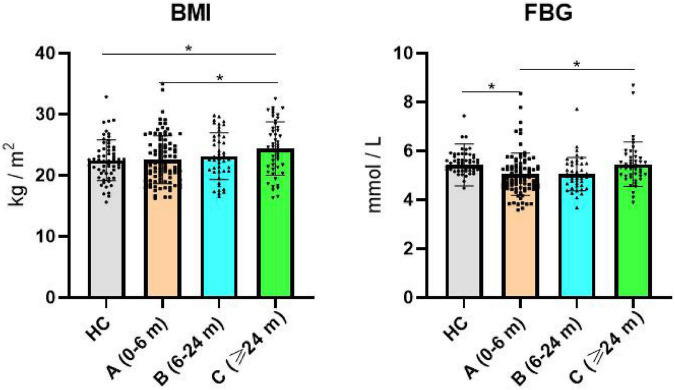
Comparison of BMI and FBG in each group. BMI, body mass index; FBG, fasting blood glucose; **(A)** schizophrenia (duration < 6 months); **(B)** schizophrenia (6 months ≤ duration <24 months); **(C)** schizophrenia (duration ≥ 24 months). *Indicates p < 0.05.

There were no significant differences in age, gender, height, weight, PANSS, HDL, LDL, TG, and TC (*p* > 0.05). Also, there were significant differences in BMI and FBG (*p* < 0.05). The incidence of obesity in groups HG, A, B, and C was 5.26, 7.61, 15.56, and 22.92%, respectively, and the incidence of overweight was 22.81, 27.17, 22.22, and 31.25%, respectively (Table 1 and Figure 1).

**TABLE 1:** Comparison of demographic characteristics and metabolic indexes.

	HC (*n* = 59)	A (*n* = 92)	B (*n* = 45)	C (*n* = 48)	*F*/χ ^2^	*P*
Course of disease (month)	–	0–6	6–24	≥ 24	–	–
Duration (month)	–	3.09 ± 1.75	11.80 ± 1.54	25.29 ± 6.35	–	–
Gender (male/female)	25/34	40/52	18/27	19/29	0.271	0.965
Age[Frame1] (year)	27.11 ± 7.76	27.09 ± 12.44	28.62 ± 12.44	25.81 ± 7.73	0.975	0.405
Education	11.61 ± 3.11	11.43 ± 2.58	11.16 ± 2.61	10.95 ± 2.80	18.91	0.091
Height (cm)	163.93 ± 7.75	164.32 ± 9.33	162.50 ± 10.22	164.38 ± 7.90	0.679	0.692
Weight (kg)	60.48 ± 10.19	60.99 ± 11.97	61.26 ± 12.31	66.15 ± 14.39	2.830	0.065
BMI (kg/m^2^)^ab^	22.47 ± 3.36	22.55 ± 3.91	23.14 ± 3.82	24.37 ± 4.37	3.247	0.040*
Obesity (%)	5.26	7.61	15.56	22.92	–	–
Overweight (%)	22.81	27.17	22.22	31.25	–	–
Non-obesity (%)	71.93	65.22	62.22	45.83	–	–
PANSS	–	98.36 ± 13.53	101.70 ± 15.51	99.35 ± 13.20	0.494	0.612
PANSS (P)	–	23.50 ± 6.64	23.51 ± 7.06	23.92 ± 5.88	0.024	0.976
PANSS (N)	–	24.70 ± 7.72	25.88 ± 7.44	24.71 ± 6.68	0.238	0.789
PANSS (G)	–	50.16 ± 6.94	52.29 ± 8.34	50.71 ± 8.80	0.683	0.508
FBG (mmol/l)^bc^	5.49 ± 0.47	5.11 ± 1.09	5.04 ± 0.68	5.57 ± 1.22	3.591	0.006*
HDL (mmol/l)	1.27 ± 0.29	1.38 ± 0.39	1.42 ± 0.50	1.36 ± 0.36	0.301	0.219
LDL (mmol/l)	2.64 ± 0.60	2.47 ± 0.74	2.62 ± 0.81	2.58 ± 0.70	0.690	0.470
TG (mmol/l)	1.12 ± 0.46	1.43 ± 1.20	1.38 ± 1.07	1.57 ± 1.12	0.341	0.132
TC (mmol/l)	4.72 ± 0.76	4.33 ± 1.02	4.59 ± 1.16	4.62 ± 0.98	1.539	0.095
Serum insulin (pmol/l)	–	13.08 ± 20.27	14.82 ± 26.73	10.85 ± 13.06	1.629	0.443

*BMI, body mass index; PANSS, positive and negative syndrome scale; FBG, fasting blood glucose; HDL, high-density lipoprotein; LDL, low-density lipoprotein; TG, triglyceride; TC, total cholesterol.*

^a^HC vs. C, ^b^A vs. C, ^c^HC vs. A, p < 0.05. *Indicates the comparison among groups (p < 0.05). Values are expressed as mean ± standard deviation.

### Pearson Correlation Between Body Mass Index and Various Indexes in Schizophrenia

The results showed that BMI was positively correlated with age, duration, FBG, LDL, TG, and TC, and negatively correlated with HDL, while PANSS, PANSS (P), PANSS (N), and PANSS (G) were not correlated with BMI (Table 2).

**TABLE 2:** Pearson correlation between BMI and various indexes in SC.

BMI								
Age*	*r*	0.314	Education	*r*	–0.028	Duration*	*r*	0.244
	*p*	<0.001		*p*	0.662		*p*	0.001
PANSS	*r*	0.045	PANSS (P)	*r*	–0.064	PANSS (N)	*r*	0.065
	*p*	0.673		*p*	0.549		*p*	0.538
PANSS (G)	*r*	0.073	FBG*	*r*	0.403	HDL*	*r*	-0.313
	*p*	0.489		*p*	<0.001		*p*	<0.001
LDL*	*r*	0.273	TG*	*r*	0.448	TC*	*r*	0.256
	*p*	<0.001		*p*	<0.001		*p*	0.001
Serum insulin	*r*	0.089						
	*p*	0.229						

*BMI, body mass index; PANSS, positive and negative syndrome scale; FBG, fasting blood glucose; HDL, high-density lipoprotein; LDL, low-density lipoprotein; TG, triglyceride; TC, total cholesterol.*

*Indicates p < 0.05.

### Multiple Linear Regression Analysis of the Influencing Factors of Body Mass Index in Schizophrenia

Taking BMI as the dependent variable (Y) and age, duration, HDL, LDL, TG, and TC as independent variables (X), and gender as a covariate, a stepwise multiple linear regression model (*F* = 16.394, *p* < 0.001) was established. Finally, the elements such as age, duration, FBG, HDL, and TG, were considered for the model (Table 3).

**TABLE 3:** Multiple linear regression analysis of influencing factors of BMI in SC.

Model	*B*	Standard error	Standard coefficient	*t*	*p*
(Constant)	16.394	1.772	−	9.254	<0.001*
Age	0.094	0.026	0.239	3.609	<0.001*
Duration	0.081	0.026	0.195	3.081	0.002*
FBG	0.987	0.341	0.260	2.899	0.004*
HDL	–2.875	0.804	–0.297	–3.576	<0.001*
LDL	0.572	0.933	–0.107	0.613	0.541
TG	0.918	0.285	0.263	3.218	0.002*
TC	0.573	0.773	0.150	0.741	0.460

BMI, body mass index; FBG, fasting blood glucose; HDL, high-density lipoprotein; LDL, low-density lipoprotein; TG, triglyceride; TC, total cholesterol. *Indicates p < 0.05.

## Discussion

Our study included first-episode, drug-naive SC with a different course of disease. By comparing their blood metabolic indexes, we found that after excluding the drug factors, the longer is the course of SC, the more obvious is the increase of BMI, and the higher is the proportion of obesity.

A previous study has shown that there was no significant difference in the incidence of obesity in first-episode SC compared with HC (13). However, after using psychotropic drugs for a period of time, the weight of patients would increase to varying degrees, even to the extent of obesity (10, 14). The abovementioned research indicated that SC itself did not cause obesity. According to a large-scale national epidemiological survey, the results show that the prevalence of obesity in urban areas in southern China was 2.8–7.2% from 2010 to 2018 (15). The course of 0–6 months of HC (5.26%) and SC (7.61%) was very close to the above range. However, some researchers believed that SC had metabolic abnormalities first, and then obesity (16). When we focused on SC patients with a longer course of disease (over 6 months), we found that the incidence of obesity was higher than the abovementioned data of the healthy population of the national epidemiological survey. Moreover, when we enrolled the subjects, most patients had a course of disease from 0 to 6 months, and fewer patients had a longer course of disease, which was also the reason that our results were consistent with those of predecessors.

Comparing the FBG of each group, we found that the FBG decreased in patients with 0–6 months course. As we know that SC always starts with negative or positive symptoms (3), psychotic symptoms can lead to eating disorders (17). When patients had positive symptoms such as victim delusion or taste hallucinations, some patients would be afraid to eat (18). They often consult doctors during the acute exacerbation period, resulting in lower blood glucose measured in the blood test the next day. With the migration of the course of disease, the patient adapted to the psychotic symptoms, and then the FBG gradually recovered. If patients started with negative symptoms, they would shrink back and need to be supervised by family members in their daily life, which would reduce food intake, resulting in the decline of FBG the next day. In addition, patients with SC with a course of more than 2 years were accompanied by undetectable mental symptoms, and the abnormal diet was not obvious (19). Therefore, there was no difference between the results of their FBG.

To further explore the factors affecting BMI, we correlated the metabolic indexes and PANSS scores with BMI. The results showed that BMI was positively correlated with age, course of disease, FBG, LDL, TG, and TC, negatively correlated with HDL, and had no correlation with the scores of PANSS in each group, which meant that the severity of SC had nothing to do with BMI. The incidence of obesity in SC increased with age, similar to most mental disorders (10, 20). At the same time, our results suggested that excluding the influence of drugs, BMI increased with the increase in the course of disease. The research team of Solmi supported our results that there were multiple risk-related genes between SC and eating behavior disorder and BMI, which were closely related to each other (21). FBG means that after fasting overnight (at least 8–10 h without any food, except drinking water), the blood sugar detected before breakfast can reflect the function of B cells in the islets, which generally indicates the secretion function of basal insulin, and is the most commonly used indicator for diabetes. FBG is closely related to BMI in the study of diabetes mellitus, which has been proved by extensive research (22, 23). LDL, TG, and TC belong to clinical blood lipid indexes. Their effects on obesity have been unanimously recognized. It is generally believed that their increase is strongly related to the occurrence of obesity (24). Finally, the results of regression analysis were further proof that age, duration, FBG, and TG were the risk factors for the increase of BMI. HDL was the protective factor.

Of course, it is best that we conduct a longitudinal follow-up study on the first-episode, drug-naive SC, and do not use any drugs to intervene, which obviously violates ethics and humanitarianism. So, we had to test this hypothesis by selecting untreated SC patients with a different course of disease. In addition, patients with a long course of disease tend to shrink back, including diet and living habits, which would lead to the deviation of our study. Overall, SC itself can increase BMI and easily lead to obesity. We should pay more attention to monitoring of blood metabolism indicators, so as to reduce the risk of obesity and improve the quality of life of patients.

## Data Availability Statement

The original contributions presented in this study are included in the article/supplementary material, further inquiries can be directed to the corresponding author/s.

## Ethics Statement

The studies involving human participants were reviewed and approved by the Ethics Committee of the Third People’s Hospital of Foshan, China. The patients/participants provided their written informed consent to participate in this study.

## Author Contributions

JL, YC, and YY made great contributions to the conception, design, and writing of the article. XX, XL, ZL, CX, and GX provided assistance in the acquisition, analysis, and interpretation of data. All authors approved the publication of the manuscript.

## Conflict of Interest

The authors declare that the research was conducted in the absence of any commercial or financial relationships that could be construed as a potential conflict of interest.

## Publisher’s Note

All claims expressed in this article are solely those of the authors and do not necessarily represent those of their affiliated organizations, or those of the publisher, the editors and the reviewers. Any product that may be evaluated in this article, or claim that may be made by its manufacturer, is not guaranteed or endorsed by the publisher.

## Footnotes

1
https://www.ibm.com/analytics/spss-statistics-software
